# 564. Patient and Prescriber Characteristics Associated with Inappropriately Long Antibiotic Duration for Skin and Soft Tissue Infections

**DOI:** 10.1093/ofid/ofac492.617

**Published:** 2022-12-15

**Authors:** Kali Broussard, Joshua R Watson, Jack Stevens, Juan D Chaparro, Mahmoud Abdel-Rasoul

**Affiliations:** Nationwide Children's Hospital, Hilliard, Ohio; Nationwide Children's Hospital, Hilliard, Ohio; Nationwide Children's Hospital/The Ohio State University Department of Pediatrics, Columbus, Ohio; Nationwide Children's Hospital, Hilliard, Ohio; Ohio State University Collect of Medicine Center for Biostatistics, Columbus, Ohio

## Abstract

**Background:**

For common infections, previous studies suggest more seasoned prescribers more commonly use inappropriately long antibiotic durations however, there is a paucity of data regarding patient and provider factors associated with antibiotic prescription duration for pediatric skin and soft tissue (SSTI).

**Methods:**

Retrospective analysis of patients age 0-21 years prescribed enteral antibiotics for cellulitis, abscess, impetigo, or other SSTI in one of 13 Nationwide Children’s Hospital’s primary care clinics in 2020. Encounters for paronychia, facial abscess, and immunocompromised patients were excluded. Based on national and local SSTI guidelines, antibiotic duration was considered inappropriately long if >5 days for cellulitis or drained abscess or >7 days for undrained abscess, impetigo, or other SSTI. Patient and prescriber factors were collected (Table 1) then univariate and multivariable analyses performed to determine which factors were associated with long antibiotic duration. Statistical analyses accounted for within prescriber correlation.

**Results:**

From 1/1/2020 to 12/31/2020, we identified 1248 encounters for SSTI. After exclusions, 577 encounters with enteral prescriptions written by 89 prescribers were analyzed. Patient age ranged from 11 days to 20 years, 314 (54%) were female, and 497 (86%) had public insurance. The most common diagnosis was impetigo in 165 (29%). Similar rates of long duration were seen across patients of all ages and insurance categories. No difference in prescriber degree type was associated with antibiotic duration. Mean years since completion of medical training for prescribers of long vs guideline concordant prescriptions was 13 vs 10 in univariate analysis (p=.001). In the multivariable analysis, only diagnosis was associated with increased odds of long duration (Table 2). Compared to impetigo, the adjusted odds ratio for inappropriately long duration was 9.36 for cellulitis and 2.03 for abscess.

Patient and Prescriber Characteristics for SSTI Antibiotic Prescriptions

**Figure:**
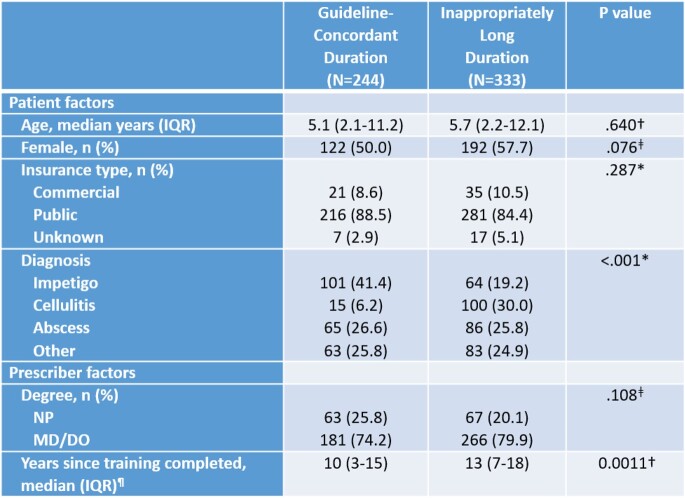

IQR, interquartile range, MD, Doctor of Medicine, DO, Doctor of Osteopathic Medicine, NP, Nurse Practitioner

†Mann Whitney ‡Fisher’s exact *Chi-square ¶N=556 due to missing data for 21 encounters

Multivariable Analysis of Patient and Provider Factors and Inappropriately Long Antibiotic Duration for SSTI

**Figure:**
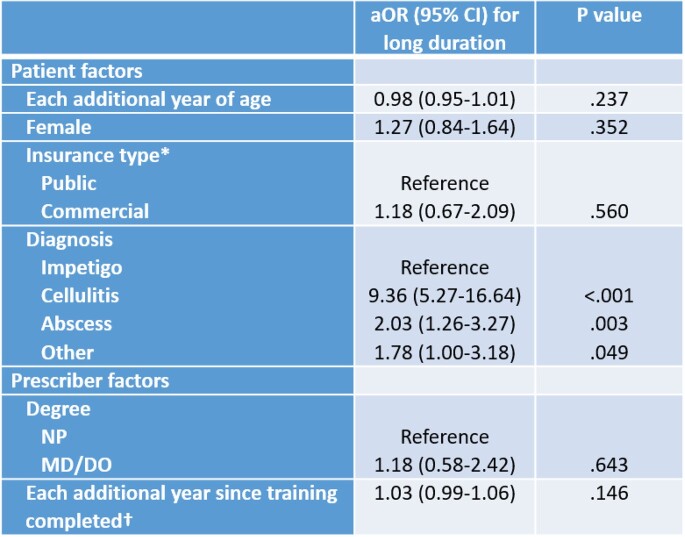

Multivariable Analysis of Patient and Provider Factors and adjusted odds ratios for inappropriately long antibiotic duration. *Unknown insurance type for 24 encounters †Missing data for 21 encounters

**Conclusion:**

In this study of antibiotic duration for SSTI, diagnosis of cellulitis was most strongly associated with inappropriately long duration- providing a focus for future stewardship efforts. Contrary to prior studies, no other patient or provider factor was associated with long duration in our pediatric primary care network.

**Disclosures:**

**Jack Stevens, PhD**, Colgate Palmolive (although this abstract does not pertain to any products/services of that highly diversified company): Stocks/Bonds|Procter and Gamble (although this abstract does not pertain to any products/services of that highly diversiified company): Stocks/Bonds.

